# The Impact of an Art Therapy Program During Antepartum Hospital Admission for High-Risk Pregnancy: Results of a Six-Month Pilot Program

**DOI:** 10.7759/cureus.78440

**Published:** 2025-02-03

**Authors:** Wendy J Smith, Tracy C Mandel, Joy M Scherzinger, Lindsay M Payne, Monica K Mitchell, Nazanine Abbaszadeh

**Affiliations:** 1 Women's Services, Legacy Health, Portland, USA

**Keywords:** anxiety and depression in pregnancy, art therapy, high-risk pregnancies, non-pharmocologic intervention, pilot program, prolonged antepartum hospitalization

## Abstract

Background and objective

Antepartum hospitalization for maternal or obstetric complications is associated with increased depression and anxiety, potentially leading to postpartum mental health challenges and elevated parenting stress. While various therapeutic interventions have shown efficacy in reducing distress associated with antepartum hospitalization, the role of art therapy has not been adequately studied in this high-risk pregnant population. This study aimed to address it and fill the gaps in the data.

Materials and methods

This prospective, single-arm intervention trial evaluated the impact of a twice-weekly art therapy program on emotional well-being and distress in hospitalized high-risk pregnant patients. Participants completed validated stress and emotional impact assessments via the Antepartum Bedrest Emotional Impact Inventory (ABEII) and the Antepartum Hospital Stressors Inventory (AHSI) before and after the intervention.

Results

Among 33 eligible patients, 22 participated in up to 15 art therapy sessions, with 15 study subjects (68%) completing a post-discharge survey. Art therapy participants reported significantly lower post-intervention stress levels compared to baseline, particularly related to separation from home and family, and expressed greater satisfaction with hospital support compared to a historical cohort.

Conclusions

Our findings suggest that art therapy may help mitigate stress and improve emotional well-being in hospitalized antepartum patients. Larger studies are warranted to confirm its therapeutic value.

## Introduction

Pregnant individuals may require hospitalization during the antepartum period due to medical conditions or pregnancy complications necessitating increased maternal and fetal monitoring and proximity to a delivery room [1,2]. The exact number of antepartum hospitalizations in the United States annually is unknown. However, a 2002 review of over 46,000 pregnancies within a national managed care organization found that 8.7% of pregnancies involved hospitalization [3]. Among these, 5.7% of patients were hospitalized and discharged while still pregnant, and 0.8% experienced extended stays before either a live birth or pregnancy loss. Recent evidence indicates that the rate of high-risk pregnancies in the United States is increasing, suggesting that antepartum hospital admissions are likely to rise in the future [4].

Prolonged hospital admissions for obstetric complications have been highly associated with depression and anxiety in pregnant individuals [1,5-7]. A 2021 meta-analysis concluded that one in three people hospitalized during the antepartum period experienced clinical levels of depression and anxiety symptoms, twice the reported prevalence of antenatal depression or anxiety in the general obstetric population [8]. Depression and anxiety during the antepartum period have been associated with decreased maternal-fetal attachment, increased risk of preterm delivery, postpartum depression, and increased parenting stress [6,9-10]. Hospitalization is inherently stressful, and antepartum admissions come with unique challenges [11]. Qualitative studies identify key stressors such as separation from home and family, boredom, lifestyle changes, concerns about maternal and fetal health, loss of control and activity, and feelings of hopelessness [12,13]. Reducing these stressors during hospitalization has been shown to alleviate stress and potentially improve associated adverse pregnancy outcomes.

Four key areas are recognized as critical for optimizing outcomes in hospitalized antepartum patients: education, support, recreation, and outreach [14]. Various non-pharmacological interventions, including cognitive behavioral therapy, pet therapy, relaxation techniques, yoga, music therapy, stress management training, support groups, and integrative therapies like acupuncture, Healing Touch, massage, and reflexology, have been studied alongside obstetric and newborn education [15-21]. These interventions are associated with significant reductions in antepartum anxiety and depression symptoms or improvements in mood perception, demonstrating their effectiveness in managing mental health in high-risk pregnant patients. Art therapy is an integrative mental health profession that enhances well-being through active art-making, creative processes, psychological theory, and human experience within a therapeutic relationship [22]. Facilitated by professional art therapists, it provides a space for meaning-making and emotional expression, often through symbolic representation, which can improve mood and reduce mental health symptoms [23,24].

Art therapy has demonstrated significant benefits in supporting the mental and emotional well-being of pregnant and postpartum individuals, particularly those experiencing uncertainty and fear about childbirth [25,26]. Additionally, it has been proven to be effective in assisting hospitalized pediatric and adult oncology patients in managing the stress of hospitalization and addressing the fear and anxiety associated with their medical experiences [27,28]. Despite these established benefits, art therapy has not been adequately and extensively evaluated as an intervention to mitigate the stressors associated with antepartum hospitalization in high-risk pregnant individuals.

Given the recognition of the importance of supporting the mental health needs of pregnant and birthing individuals, a gap remains in the literature regarding the benefits of art therapy for hospitalized high-risk antepartum patients. This study aims to evaluate the effectiveness of art therapy in reducing stress symptoms associated with prolonged hospitalization during pregnancy. We hypothesize that implementing an art therapy intervention for high-risk antepartum patients admitted to the hospital for at least 72 hours will improve scores on validated measures of emotional impact and psychosocial distress, thereby decreasing their perceived stress levels during hospitalization.

## Materials and methods

We conducted a prospective, single-arm intervention trial at our level IV maternal care unit. High-risk pregnant patients are admitted to our family birth unit for days to months for maternal and fetal monitoring before delivery or discharge for continuation of care in the outpatient setting. All antepartum patients hospitalized from January 1 through June 27, 2023, were identified on the first or second day of their admission. If they remained hospitalized on day three or four with no plan for immediate discharge or delivery, the patient was offered enrollment in the study with planned participation in art therapy sessions within three days of enrollment. The exclusion criteria were as follows: inability to either read or speak English or Spanish, not having access to an email address, and medical instability not allowing participation in art therapy in either a group or individual setting. The patients signed a consent form with a trained investigator.

Each participant completed a baseline 65 short-answer question survey comprising Antepartum Bedrest Emotional Impact Inventory (ABEII) and Antepartum Hospital Stressors Inventory (AHSI) (Appendix). The ABEII is a brief assessment tool developed and validated for use in high-risk obstetric patients to measure bedrest-related psychosocial distress [20]. It includes 18 items dealing with anxiety, loneliness/isolation, boredom, depression, stress, and loss of control. The score from each item is summed up to create a total antepartum distress score (value range: 18-90), with a higher score indicating increased distress. The AHSI is a validated assessment tool that attempts to elucidate the impact of stressors that may be experienced by high-risk pregnant people hospitalized for obstetric complications [29]. It uses numbered Likert scales to evaluate 47 potential stressors assigned to seven major categories: separation, environment, health status, communications with health professionals, self-image, emotions, and family status. The range of values for the total AHSI score is 0-188, and each sub-category score ranges from 0 to 20 to 0 to 36. For both the total AHSI score and each sub-category within the AHSI, a higher score is associated with a greater impact from these stressors.

Following enrollment, each participant met with an art therapist, a professional with a master’s degree in art therapy and counseling who was registered with the National Art Therapy Association, twice weekly until hospital discharge with an ongoing pregnancy or until their delivery. The art therapist used a flexible, patient-centered approach to support the participants, offering choices in materials, themes, and structure to empower them and facilitate self-expression through art. Sessions followed an open studio model, allowing participants to process fears, isolation, and hopes for the future while fostering a sense of control, connection, and emotional validation, with group interactions providing additional peer support. Participants created paintings, mixed-media collages, and keepsake boxes among other art pieces.

One week following hospital discharge, each participant received an email with a link to an electronic survey with 65 short-answer questions identical to the baseline survey. Patients were asked to consider how they felt while they were hospitalized in answering each of the questions. Participants who did not return their survey within four days received two additional electronic reminders four days apart. Each participant's baseline ABEII and AHSI scores were compared to that individual’s post-intervention scores; these paired scores were then evaluated for significant differences using a nonparametric, one-sided Wilcoxon signed-rank test. The significance level was set at 0.05. Characteristics of participants who did not return their post-intervention survey were compared to those who did return their survey with a two-sample t-test. The post-intervention survey additionally asked participants to rate their satisfaction with their overall hospital stay on a scale of 1 to 10. The mean level of satisfaction from study participants was compared to that of a historical cohort of discharged patients who had been hospitalized during their pregnancy but did not participate in any type of activity program. All analyses were performed using Microsoft Excel 2021. The Institutional Review Board of Legacy Health, Portland, OR approved this study.

## Results

**Figure 1:**
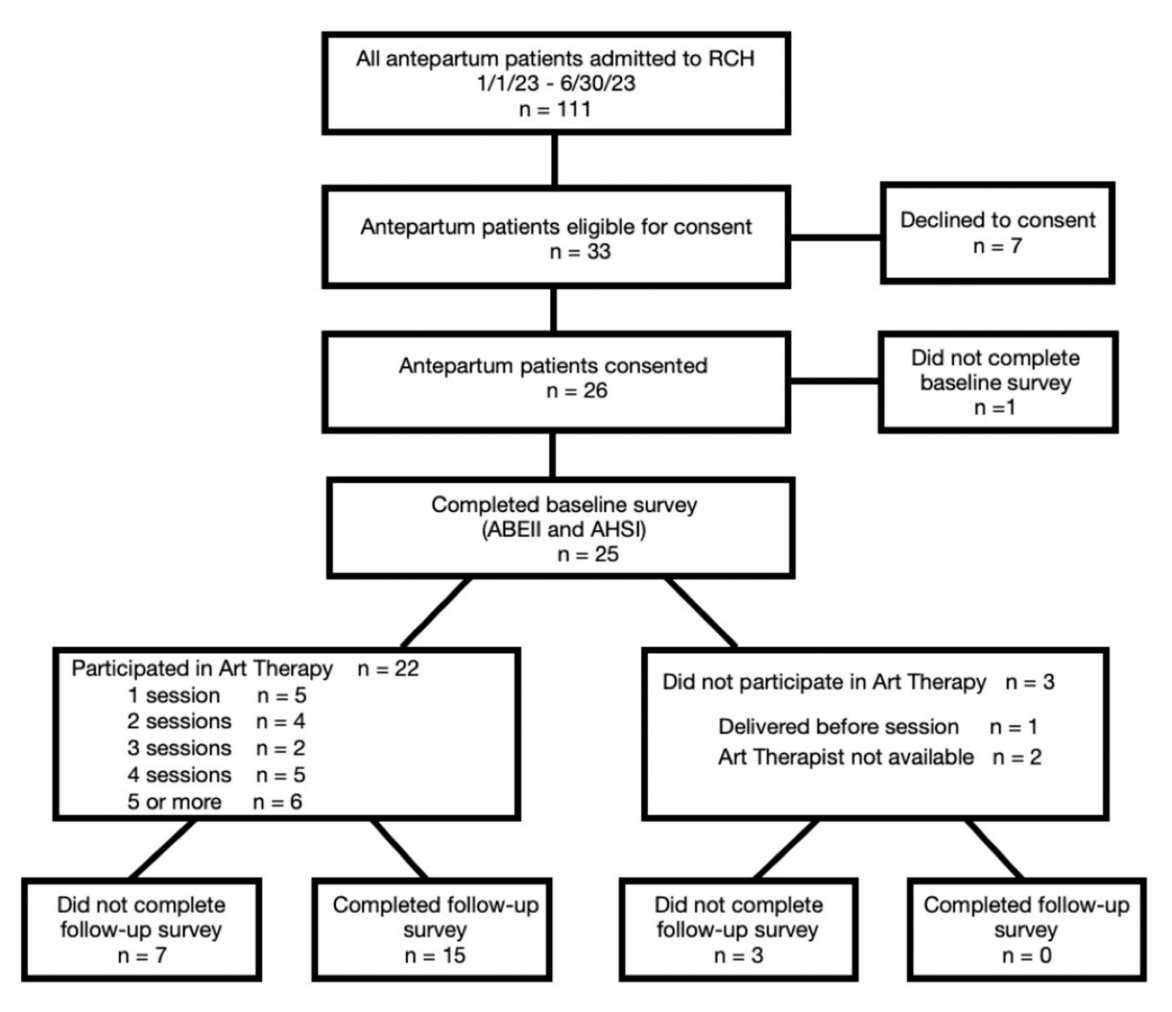
Identification, enrollment, and participation of the study subjects Flow diagram detailing the identification and enrollment of hospitalized antepartum patients during the six-month pilot. Post-discharge response rate: 60%. Response rate if the patient had participated in at least one art therapy session: 68% ABEII: Antepartum Bedrest Emotional Impact Inventory; AHSI: Antepartum Hospital Stressors Inventory

Over the six-month pilot program, 111 antepartum patients were admitted to the Family Birth Unit. Of these, 33 were eligible to participate in the study and 26 provided consent. Due to either an unanticipated delivery or the absence of the art therapist with illness, a total of 22 pregnant individuals took part in at least one and up to 15 art therapy sessions. Fifteen patients who had participated in art therapy completed an electronic post-intervention survey; seven patients did not return the electronic post-intervention survey (Figure 1).

Subjects who did not complete their post-intervention survey were more likely to live far from the hospital and had previously had more deliveries at the time of study enrollment (Table 1). Additionally, they had a significantly higher baseline mean ABEII distress score (42.2 vs. 33.3, p=0.036), and their infants spent a greater number of days in the neonatal intensive care unit (NICU) after delivery than those who completed their post-intervention survey (Table 2). Participants who returned their post-intervention survey had been hospitalized for a significantly higher number of days compared to participants who failed to return the electronic survey (20.9 days vs. 13.3 days, p=0.044) and they trended towards having completed a greater number of art therapy sessions than those who did not return their post-intervention survey [4.3 (range: 1-15) vs. 2.1 (range 0-6), p=0.052)].

**Table 1:** Sociodemographic characteristics of pilot participants Sociodemographic characteristics of participants in the art therapy pilot study stratified by whether or not they returned their electronic post-intervention survey. Characteristics of participants who did not return their post-intervention survey were compared to those who returned their survey with a two-sample t-test.

	Did not complete follow-up survey (n=10)	Completed follow-up survey (n=15)	t statistic, p-value
Maternal age, years, mean	29.2	32.3	t(23)=-1.53, p=0.070
Race/ethnicity (%)			
White/European	40	60	
Black or African American	0	6.7	
Latina or Hispanic	60	26.7	
Asian	0	6.7	
Employment status, %			
Unemployed	60	53.3	
Employed	40	46.7	
Insurance, %			
Private	30	46.7	
Medicaid	70	53.3	
Distance from hospital, miles, mean	68.5	30.3	t(23)=2.33, p=0.015
Gravidity, mean	3.3	2.7	t(23)=0.69, p=0.248
Parity, mean	1.6	0.7	t(23)=1.37, p=0.092
Other children living at home, %	60	46.7	
Family or partner involved, %	100	100	

**Table 2:** Pregnancy, hospitalization, and clinical characteristics of pilot participants Pregnancy, hospitalization, and clinical characteristics of participants in the art therapy pilot study stratified by whether or not they returned their electronic post-intervention survey. Characteristics of participants who did not return their post-intervention survey were compared to those who returned their survey with a two-sample t-test ABEII: Antepartum Bedrest Emotional Impact Inventory; ACD: advanced cervical dilatation; AHSI: Antepartum Hospital Stressors Inventory; NICU: neonatal intensive care unit; PPROM: preterm premature rupture of membranes; PTL: preterm labor

	Did not complete follow-up survey (n=10)	Completed follow-up survey (n=15)	t statistic, p-value
Gestational age at admission, weeks, mean	26.7	29.2	t(23)=-1.60, p=0.062
Fetal number, %			
Singleton	90	60	
Twins	0	40	
Triplets	10	0	
Admission diagnosis, %			
PPROM	40	33.3	
PTL/ACD/CI	20	26.7	
Preeclampsia	20	13.3	
Vasa previa	0	13.3	
Placenta previa, vaginal bleed	10	13.3	
Fetal arrhythmia, TTT, other fetal	40	0	
Total hospital days, mean	13.3 (range 4-22)	20.9 (range 7-56)	t(23)=-1.78, p=0.044
Delivered, %	80	86.7	
Discharged w/o delivery, %	20	13.3	
Gestational age at delivery or discharge, weeks, mean	28.7	32.1	t(23)=-1.69, p=0.052
NICU admission, %	100 (10/10)	88.9 (16/18)	
Total NICU days until discharge or demise	53.1 (range 1-100)	39.4 (16-125)	t(23)=0.72, p=0.241
Neonatal demise, %	30.0 (3/10)	10.5 (2/19)	
Baseline ABEII, mean (range: 18-90)	42.2	33.2	t(23)=1.88, p=0.036
Baseline total AHSI, mean (range: 0-188)	85.9	78.5	t(23)=0.641, p=0.264
Number of art therapy sessions	2.1 (range 0-6)	4.3 (1-15)	t(23)=-1.69, p=0.052

**Figure 2:**
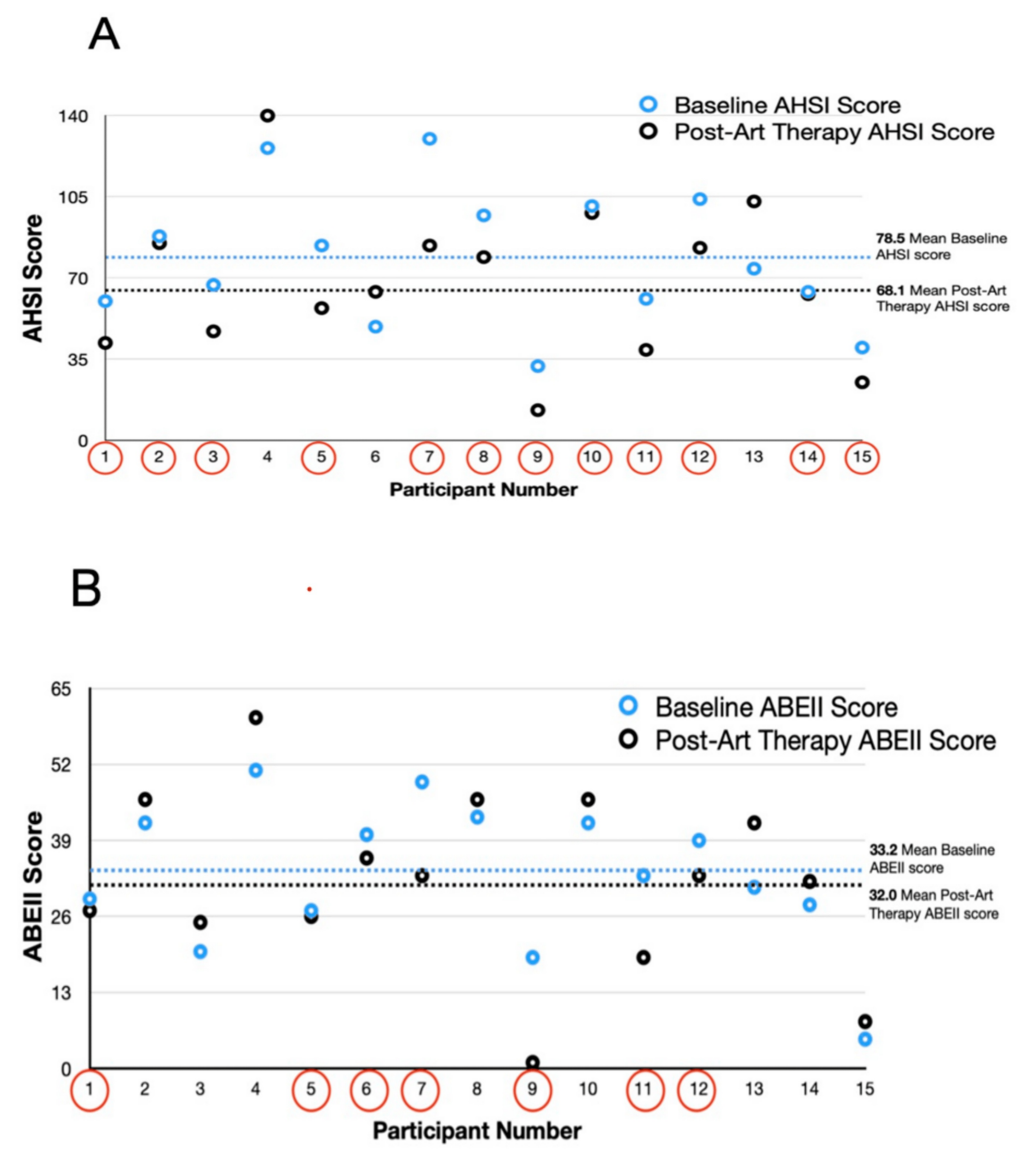
Measuring the impact of art therapy on AHSI and ABEII distress scores For each instrument, a higher score is associated with a greater level of distress. Those participants who are circled showed a lower score post-intervention than at baseline. A nonparametric, one-sided Wilcoxon signed-rank test was used to examine differences between paired scores from baseline to post-intervention; the mean difference was assessed for significance (⍺=0.05). A: Baseline total AHSI score compared with post-intervention total AHSI score for each study subject who completed a post-intervention survey (n=15). The range of possible score on the AHSI is 0-188. The mean AHSI score following art therapy was significantly lower than the mean baseline score for all participants (p=.027). B: Baseline ABEII score compared with post-intervention ABEII score for each study subject who completed a post-intervention survey (n=15). The range of possible score on the ABEII is 18-90. The mean ABEII score following art therapy was not significantly different than the mean baseline score for all participants (p=0.304) ABEII: Antepartum Bedrest Emotional Impact Inventory; AHSI: Antepartum Hospital Stressors Inventory

Hospitalized antepartum pregnant patients who participated in art therapy sessions and returned their post-intervention survey showed a significantly lower mean level of impact from stress on their post-discharge stress score than that at baseline on the AHSI (Figure 2A). The mean post-discharge ABEII score was not found to be significantly different from that at baseline in art therapy participants; however, seven out of 15 respondents did show lower emotional distress scores at follow-up (Figure 2B). While art therapy participants had significantly lower values of stress related to separation from home, work, and family on this AHSI sub-scale, none of the other sub-scales were associated with a significant difference from the baseline value. Art therapy participants who returned their post-intervention survey reported a significantly higher mean level of satisfaction (based on a 10-point Likert scale) with their overall hospital stay compared to a 2022 historical baseline mean value reported by 37 hospitalized antepartum patients before the art therapy pilot (9.2 vs. 6.4, p<0.05).

## Discussion

Prolonged hospitalization during pregnancy can lead to distress in antepartum patients. Our results suggest that providing support to this cohort of hospitalized antepartum patients via art therapy is associated with a significant reduction in the overall impact of the stressors related to antepartum hospitalization, and may be most effective at alleviating distress associated with separation from patients’ usual relationships and activities. The results from our pilot study additionally suggest that some patients may benefit from art therapy more than others, including those with the most prolonged hospitalizations, those who participate in a greater number of art therapy sessions, and those who live farthest from the hospital, a possible proxy for having fewer family and friends available for support.

Current evidence suggests that art therapy benefits pregnant and postpartum individuals. For instance, low-risk pregnant individuals with severe fear of childbirth experienced increased hope and self-confidence after six art therapy sessions, which correlated with a reduced rate of cesarean deliveries compared to psychoeducation alone [25]. Hogen et al. reviewed the use of art therapy among recent postnatal mothers and found that it enhanced self-esteem, improved parental self-image, and facilitated bonding between mother and infant [26]. Additionally, art therapy has shown efficacy in promoting healing, managing stress, and alleviating symptoms in hospitalized populations, particularly adult cancer and pediatric patients [27,28].

Our study adds to the limited literature evaluating the role of art-making for hospitalized pregnant people. Only two published studies have specifically assessed the use of art in this population so far. Bauer et al. found that antepartum inpatients who participated in “independent craft activities” for one hour had an associated reduction in distress lasting 48-72 hours [16]. Lee et al. performed a qualitative analysis of the benefits of an art therapy program for 49 hospitalized women experiencing high-risk pregnancies and published their work in abstract form only [30]. Their study found that “art therapy gave [hospitalized women] a creative way to cope with their stress, grieve loss, find meaning, and have a normalizing experience in an abnormal environment.” 

An important limitation of our study that may affect its generalizability is the small number of eligible participants. Unfortunately, the timeline of our pilot coincided with the closure of one of our organization’s nearby low-risk labor and delivery units. The majority of patients planning to deliver at that unit were referred to our Level IV unit for their labor. This led to space and staffing issues, which resulted in the diversion of antepartum patients requiring admission to a different hospital. Comparing our census during the six-month pilot with a historical antepartum admission census for the same six-month period one year earlier showed that we had 20 fewer admissions (111 vs. 131) and 19 fewer eligible patients (52 vs. 33). Our low participation numbers may have contributed to the failure to identify a significant decrease in ABEII score at baseline and post-intervention, as well as selection bias; it is possible that patients who participated in the pilot were less likely to be impacted by art therapy than others might have been.

Additionally, the limited sample size prevented us from conducting meaningful regression analysis to identify characteristics of patients most likely to benefit from art therapy, as well as determining the minimum number of sessions needed for an effect. The limited number of eligible participants also made performing a possibly more informative double arm or randomized (to "usual care" or no art therapy) trial infeasible in our system. Another limitation is that our study could not assess how follow-up survey scores may be impacted by several external factors including poor support after discharge, a death or critical condition of the neonate, and postpartum maternal medical complications. Additionally, our study did not evaluate whether participants who have baseline mental health or behavioral health disorders are differentially impacted by participating in art therapy during their hospitalization. Finally, this pilot study evaluated only the short-term impact of art therapy on stressors affecting hospitalized pregnant patients' mental health, without assessing longer-term mental health outcomes or effects on parenting.

## Conclusions

When recommending antepartum hospitalization, physicians must consider the potential impact of prolonged admission on a pregnant patient's mental health. Our study suggests that art therapy may alleviate certain types of distress associated with extended antepartum hospitalization. To comprehensively assess the efficacy of inpatient art therapy programs in mitigating hospitalization-related distress among antepartum patients, future studies should incorporate larger sample sizes, a double-arm design, and extended follow-up periods. This approach would facilitate a thorough evaluation of both the short-term and long-term effects of art therapy on maternal mental health and parenting stress, thereby elucidating its full spectrum of potential benefits.

## Appendices

**Table 3:** Antepartum Bedrest Emotional Impact Inventory (ABEII) Instructions: Please CIRCLE one response in the right column to each statement in the middle column that best matches how you feel while you are (or were) in the hospital during your pregnancy

	While in the Hospital on Bedrest During My Pregnancy…	
1	I feel bored	1 = Never
		2 = Hardly Ever
		3 = Sometimes
		4 = Often
		5 = Always
2	I feel anxious	1 = Never
		2 = Hardly Ever
		3 = Sometimes
		4 = Often
		5 = Always
3	I feel inadequate	1 = Never
		2 = Hardly Ever
		3 = Sometimes
		4 = Often
		5 = Always
4	I cry more than usual	1 = Never
		2 = Hardly Ever
		3 = Sometimes
		4 = Often
		5 = Always
5	I feel stressed	1 = Never
		2 = Hardly Ever
		3 = Sometimes
		4 = Often
		5 = Always
6	I feel out of control	1 = Never
		2 = Hardly Ever
		3 = Sometimes
		4 = Often
		5 = Always
7	I feel sad or depressed	1 = Never
		2 = Hardly Ever
		3 = Sometimes
		4 = Often
		5 = Always
8	I feel that I have very few options	1 = Never
		2 = Hardly Ever
		3 = Sometimes
		4 = Often
		5 = Always
9	I feel that I have little to look forward to each week	1 = Never
		2 = Hardly Ever
		3 = Sometimes
		4 = Often
		5 = Always
10	I find it hard to keep my mind off my concerns about my pregnancy	1 = Never
		2 = Hardly Ever
		3 = Sometimes
		4 = Often
		5 = Always
11	I get sudden feelings of panic	1 = Never
		2 = Hardly Ever
		3 = Sometimes
		4 = Often
		5 = Always
12	I find it hard to control the minor things that bother me in my life	1 = Never
		2 = Hardly Ever
		3 = Sometimes
		4 = Often
		5 = Always
13	I find it hard to keep my emotions steady or balanced	1 = Never
		2 = Hardly Ever
		3 = Sometimes
		4 = Often
		5 = Always
14	The time passes very slowly for me	1 = Never
		2 = Hardly Ever
		3 = Sometimes
		4 = Often
		5 = Always
15	I feel very alone	1 = Never
		2 = Hardly Ever
		3 = Sometimes
		4 = Often
		5 = Always
16	I feel angry because things are happening that are outside my control	1 = Never
		2 = Hardly Ever
		3 = Sometimes
		4 = Often
		5 = Always
17	I feel depressed or sad about being less active than usual	1 = Never
		2 = Hardly Ever
		3 = Sometimes
		4 = Often
		5 = Always
18	I have a hard time relaxing	1 = Never
		2 = Hardly Ever
		3 = Sometimes
		4 = Often
		5 = Always

**Table 4:** Antepartum Hospital Stressors Inventory (AHSI) Instructions: People who are pregnant and must stay in the hospital find they have to cope with many new things. These things can be upsetting or stressful to them. We usually think of stress as something that makes it difficult for us to relax. Here is a list of experiences which pregnant people who have to stay in the hospital might find stressful. Please check the box in the column that describes the amount of stress you feel when you think about the statement on the left side

		No Stress	Very Little Stress	Some Stress	A Lot of Stress	A Great Amount of Stress	Does Not Apply to Me
1	Being less active than usual						
2	Wanting to be home to get ready for the baby						
3	Taking medication						
4	Thinking about my health						
5	Understanding what tests mean and why they are done						
6	Sleeping alone						
7	Being away from my work						
8	Thinking about being a mother						
9	Having tests done						
10	Being away from home						
11	Being asked about myself by other patients and their visitors						
12	Trying to understand medical terms						
13	Being given too much information about my condition						
14	Thinking about my baby’s health						
15	Feeling worried						
16	Sleeping in a strange bed						
17	Being dependent on others						
18	Thinking about my partner doing my work						
19	Being away from my partner						
20	Lacking privacy						
21	Feeling scared						
22	Being away from my usual activities						
23	Thinking about the care of my children at home						
24	Feeling depressed						
25	Being bored from lack of activities						
26	Having hospital prepared meals						
27	Wondering how long I will be in the hospital						
28	Hearing heartbeats on monitors						
29	Missing prenatal classes						
30	Feeling angry						
31	Being dressed for bed all the time						
32	Being isolated from my friends						
33	Being given too little information about my condition						
34	Thinking about extra expenses while I am in the hospital						
35	Hearing the staff be noisy						
36	Thinking about other patients’ heath						
37	Having nurses check my baby’s heart rate						
38	Depending on staff to keep my room clean						
39	Hearing hospital noises						
40	Sharing a room with another patient						
41	Noticing staff are hurrying with my care						
42	Thinking about giving birth						
43	Needing a special diet						
44	Thinking about the results of tests						
45	Telling unfamiliar staff about myself						
46	Feeling lonely						
47	Being away from my family						
